# The effect of UV-protected ethylene vinyl acetate (EVA) bags on the physicochemical stability of pediatric parenteral nutrition admixtures

**DOI:** 10.1007/s40199-019-00270-7

**Published:** 2019-05-17

**Authors:** Dorota Watrobska-Swietlikowska, Ronan MacLoughlin

**Affiliations:** 1 Department of Pharmaceutical Technology, Medical University of Gdansk, Hallera Av. 107, 80-416 Gdansk, Poland; 2 Aerogen, IDA Business Park, Dangan, Galway, Ireland; 3 School of Pharmacy, Royal College of Surgeons, Dublin, Ireland; 4 School of Pharmacy and Pharmaceutical Sciences, Trinity College, Dublin 2 Dublin, Ireland

**Keywords:** Pediatric parenteral nutrition, Stability of vitamins, UV-protected bags, Ethylene vinyl acetate bags, Physicochemical stability

## Abstract

**Background:**

The safe administration of parenteral admixtures should be considered under the headings of physical and chemical stability. Vitamins are considered to be most susceptible to chemical degradation.

**Objectives:**

To evaluate the protective effect of UV-protected monolayer ethylene vinyl acetate (EVA) bags in comparison with that of EVA bags without UV protection, on the physicochemical characteristics and stability of the light sensitive vitamins in pediatric parenteral admixtures stored under various temperature and light conditions.

**Methods:**

Four different parenteral pediatric admixtures (with trace elements and vitamins) in two types of ethylenovinylacetate (EVA) monolayer containers (with – yellow one and without – transparent one UV protection) were assessed. The physicochemical analyses such as visual inspection, pH and potential zeta measurements, lipid globules size distribution and vitamins concentration were performed at 0 h, 24 h, 8 days and 8 days+24 h after the preparation of the TPN admixtures. In order to quantify ascorbic acid, thiamine and pyridoxine levels, HPLC was used.

**Results:**

No differences (*p* < 0.05) in physicochemical stability of TPN admixtures were noted between two types of EVA bags, with the compositions assessed; stored 8 days (4 °C ± 2) without light plus 24 h at room temperature and light exposure. However significant differences were noticed in ascorbic acid, thiamine and pyridoxine content after 8 days+24 h in comparison with t = 0. This was noted for both for UV-protected bags and bags without UV-protection, Nevertheless, amounts were still within the pharmacopeial range.

**Conclusions:**

Both EVA bags under test (with and without UV-protection) ensure physicochemical stability up 8 days at 4 °C ± 2 °C without light exposure and then 24 h at room temperature with light exposure for the total pediatric parenteral admixtures, intended for home parenteral nutrition.

**Graphical abstract:**
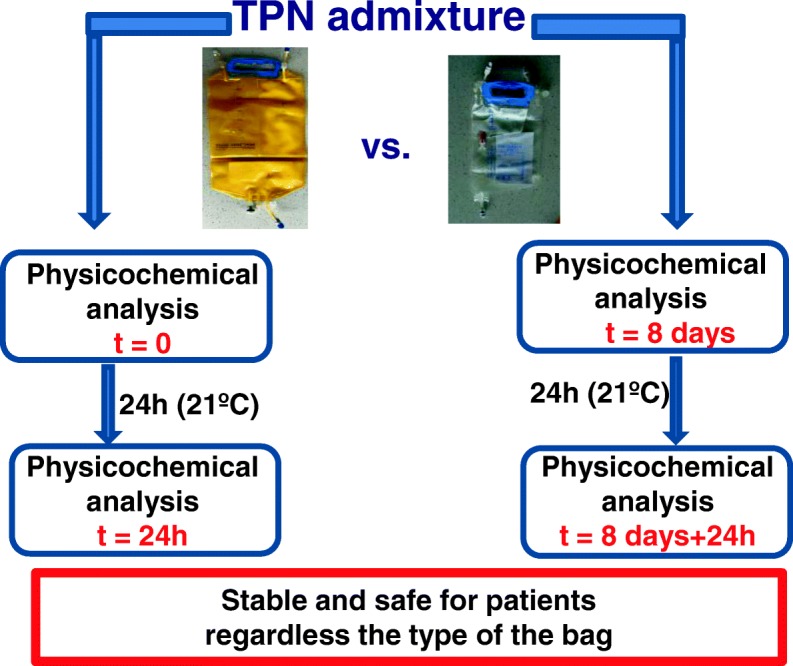
Scheme of physicochemical analysis of parenteral admixtures

## Introduction

Parenteral nutrition is one of the most complex drug preparations used in modern medicine. Detailed analysis of the nutritional mixtures indicate that almost 50 different components may be mixed together and stored in a single bag. Such complexity increases the chances of interactions, both between components, and between components and the packaging material [1, 2].

Problems of compatibility and stability can occur particularly in total parenteral nutrition (TPN) admixtures prepared for, and administered to premature infants. This is largely due to the resulting higher concentrations of nutrients per unit volume in the reduced final volume necessary for neonatal admixtures [3, 4]. Proper planning of parenteral nutrition in children is a significant challenge, and much more complex than parenteral nutrition in adult patients [5, 6]. Further, neonate TPN admixtures are characterized by a high proportion of carbohydrates, amino acids and lipids, as neonates have higher energy requirements than older children or adults [7, 8].

Regardless of the level of inherent stability, vitamins are considered to be most susceptible to chemical degradation [1, 9]. The most common and important reaction is oxidation of ascorbic acid and the reduction of thiamine [10]. Vitamins regulate growth and support the use and storage of energy [11]. Inactivation of vitamins may follow many mechanisms: photolysis of vitamin A and B1, oxidation of vitamin C, reduction of vitamin B1 or adsorption of vitamin A onto the surface of the container [12, 13]. An important consideration is that stability testing of vitamins should be carried out, at a minimum, during the infusion period and under the same ambient conditions (light and room temperature).

Hospital pharmacists are presented with many challenges when it comes to the addition of vitamins and trace elements to TPN admixtures. For example some authors suggest that vitamins and trace elements should not be added to the same admixture due their incompatibilities and instabilities [9]. It is well known that the least stable water-soluble vitamin is ascorbic acid, mainly due its oxidation properties. This reaction is catalyzed by bivalent ions, especially copper. Ascorbic acid is also involved in the reduction of the selenite ion to elemental selenium, and that has the potential to form precipitates [14]. Due to lack of specific evidence [11] on the compatibility of trace elements with other vitamins, some authors suggest approaches such as administration of vitamins and trace elements via two separate but concurrent intravenous administrations [1]. Supplementation of neonates can be further complicated as continuous administration of all components is required, and over extended periods. In many hospitals the addition of vitamins and trace elements to the TPN admixture occurs immediately before the administration. On the other hand, vitamins play a role as a protectant from peroxidation for lipid emulsions, thereby suggesting that vitamins should be added together with other excipients to parenteral admixtures [9].

Stability of TPN admixtures should be considered under the headings of physical and chemical stability. The most critical parameter is the size of lipid emulsion droplets. Lipid emulsion droplets should be below the size of smallest blood vessels in order to mitigate the risk of embolism, which presents a significant risk for the patient [15]. Lipid emulsion droplet size and the associated size distribution assessments should be carried out as a basic analysis of TPN admixture stability, and in combination with electrophoretic mobility (zeta potential) which guarantees the kinetic stability of the lipid emulsion [16].

The objective of this study was to quantify light sensitive vitamins: ascorbic acid, thiamine and pyridoxine in pediatric total parenteral nutrition admixtures intended for home parenteral nutrition in order to investigate the influence of UV-protection monolayer bags on degradation of these vitamins in pediatric admixtures. A second aim of this study was to evaluate the effect of the level of UV protection provided by the bag on the physical stability of the admixtures through characterisation of the droplet size distribution, average droplet size, zeta potential and pH measurements.

## Methods

### Composition and preparation of TPN admixtures

During the study, four different TPN admixtures (complete with trace elements and vitamins) (I-IV) in two types of ethylene vinyl acetate (EVA) monolayer containers (with – yellow one and without – transparent one UV protection) were assessed. The composition of these admixtures are representative of those commonly used in clinical practice at hospital we collaborate with. The maximum concentration of nutrients was adopted here in an effort to represent the worst case scenario. A detailed composition of these TPN admixtures and their osmolarity is presented in Table 1. All TPN admixtures were prepared aseptically under a laminar airflow hood in a clean room, following international guidelines.

**Table 1:** Composition and osmolarity of TPN admixtures under test

Ingredient	Preparation	Unit	I	II	III	IV
Amino acids	Vamin 18 Electrolyte Free	[ml]	90	350	–	–
Amino acids	Primene 10%	[ml]	–	–	80	–
Amino acids	Aminoven Infant 10%	[ml]	–	–	–	60
Carbohydrates	Glucosum 20%	[ml]	270	1000	200	180
Lipid emulsion	ClinOleic 20%	[ml]	20	95	30	–
Lipid emulsion	SMOFlipid	[ml]	–	–	–	20
Sodium	Natrium chloratum 10%	[mmol]	55.52	160	31.23	27.81
Potassium	Kalium chloratum 15%	[mmol]	30	86	10	8
Calcium	Calcium glucobionas 6%	[mmol]	3.6	12	2.4	3.4
Magnesium	Magnesium sulfuricum 20%	[mmol]	0.24	2.4	1.2	0.4
Phosphates	Glycophos	[mmol]	1.15	12	2	2
Trace elements	Peditrace	[ml]	5	10	10	3
Vitamins	Soluvit N	[ml]	10	10	10	5
Vitamins	Vitalipid N Infant	[ml]	10	10	10	5
Total volume		[ml]	568.3	2033	456	470.5
Osmolality		[mOsm]	1019	1030	879	741

Monolayer ethylene vinyl acetate (EVA) bags (transparent), type Freka were obtained from Fresenius Kabi, Sweden; UV-protected monolayer ethylene vinyl acetate (EVA) bags (type MIB 1000 3 K) (yellow bags) were obtained from Hegewald Mediyinprodukte GmbH, Germany. These UV-protected bags are manufactured incorporating UV-blocking additives in the polymer material.

TPN admixtures components included the following: SMOFlipid, (Fresenius Kabi, Austria) or ClinOleic (Baxter, Belgium), Vamin 18 Electrolyte-free solution for infusion (Fresenius Kabi, Sweden), Aminoven 10% Infant (Fresenius Kabi, Sweden) or Primene 10% (Baxter, Belgium); Glucose 20% solution (B. Braun, Germany); Magnesium sulfate 20% solution (Polpharma, Poland); Potassium chloride solution 15% (WZF Polfa, Poland); Sodium chloride solution 10% (Polpharma, Poland); Calcium Pliva 10% - solution of calcium gluconolactobionate (glubionate) (Pliva, Poland); Glycophos – Sodium glycerophosphate concentrated solution (Fresenius Kabi, Sweden); Peditrace - mixture of trace elements, concentrated solution (Fresenius Kabi, Sweden) and multiple vitamin preparations - Vitalipid N Infant lipid emulsion (Fresenius Kabi, Sweden) and Soluvit N lyophilisate for solution (Fresenius Kabi, Sweden).

### Storage and sampling

All of the prepared TPN admixtures were studied both with and without UV protection (A – without UV protection, transparent bag, B –UV protected, yellow bag). Vitamins were added de novo with other components. Samples for analysis were taken aseptically from each admixture at appropriate intervals. Three technical replicates of each TPN admixture were prepared.

The first group of TPN admixtures (I-IV), were prepared in both without UV protection (samples A) and in UV-protected bags (samples B), and were assessed immediately after preparation (t = 0). All admixtures were assessed again after 24 h of storage at room temperature, under regular light conditions (t = 24 h).

A second group of samples (I-IV), prepared both bags without UV protection (samples A) and UV-protected bags (samples B), was stored for 8 days (t = 8 days) in a refrigerator without light, at 4 ± 2 °C. All admixtures were stored for 2 h at room temperature (21 ± 2 °C) prior to assessment. After sampling (t = 8 days), admixtures were stored for the next 24 h at room temperature (21 ± 2 °C), exposed to ambient light without extra light protection, and analyzed again (t = 8 days +24 h).

### Evaluation of physicochemical stability

Physicochemical analyses was performed at 0 h, 24 h, 8 days and 9 days after the compounding the parenteral admixtures. The first analysis, time 0, was undertaken 12 h after compounding, accounting for the time taken for transportation of the admixtures, under controlled conditions (4 °C), from the hospital to our department where the analysis took place. Before analysis, each TPN admixture was incubated for 2 h at room temperature, and at ambient light levels. Analysis was performed with three different aliquots of each parenteral admixture under test. To guarantee the homogenization of admixture before the sampling for analyses, the TPN bags were gently agitated. Visual observations were assessed for creaming, phase separation and color alteration.

### pH measurement

The pH measurement of parenteral admixtures was carried out in triplicate (*n* = 9 for each composition). The pH meter (Orion 350, Beverly, USA, with combination electrode) was calibrated with buffer solutions across the range; pH 4.0 and 7.0. 8 mL of parenteral admixtures were used for each measurement. The pH measurements were made at room temperature (21 °C) by direct immersion of the electrode in the admixture.

### Determination of lipid droplet size

The optical microscopy, dynamic light scattering and laser diffractometry was used to determine the size of oil globules. The size and microscopic characteristics of the oil globules was determined using an optical microscope with connected camera (B1 223A Motic, Wetzlar, Germany). This method facilitates visualization of the droplet size above1 μm without any dilution of the admixture. 40-fold magnification was applied. Microscopy samples were inspected bacross five individual visual fields. The amount of oily droplets and their diameters were measured and the microscopic images were transferred to the software Multiscan and photographs of the parenteral admixtures were taken and documented.

The droplet size of emulsions was determined using dynamic light scattering (DLS), which covers a size range of 20 to 5000 nm and uses a helium-neon laser light and an integrated analysis software (Zetasizer Nano ZS model ZEN 3600, Malvern Instruments, Malvern UK). Each sample was examined in triplicate at 21 °C (*n* = 9 for each composition). Data are shown in terms of effective mean diameter (Z-average) and the polydispersity index (PI), which reflects the width of the droplet size distribution. Prior to analysis, the samples of parenteral admixtures were collected with sterile syringes and needles and diluted with water for injection 1:100. Each analysis was performed in triplicate (*n* = 9 for each composition).

The second droplet sizing technique employed was laser diffractometry (LD, MasterSizer E, Malvern Instruments, Malvern, UK). All results were measured using the monomodal model and calculated according to the Mie theory. 10 mL of parenteral admixture was directly transferred to 500 mL of water for injection in a beaker with a stir bar. Data were transferred from Mastersizer software for calculation the volume diameters D_0.5_ and D_0.9_ which means 50% and 90% or all of the particles are below the given size.

### Analysis of zeta potential

Zeta potential was determined by microelectrophoresis using a Zetasizer Nano ZS (Malvern Instruments, United Kingdom). The Zetasizer measured the velocity of oil globules which were moved in an applied electric field. Zeta potential, expressed in mV, was inferred from electrophoretic mobility, using the Smoluchowsky formula. Measurements were carried out at 21 ± 2 °C. Each TPN admixture was analysed in triplicate (*n* = 9 for each composition). Before analysis the samples were diluted 1:100 with water for injection. Between each measurement, the microelectrophorectic cell was rinsed with water for injection.

### Quantitative analysis of ascorbic acid, thiamine and pyridoxine

**Fig. 1:**
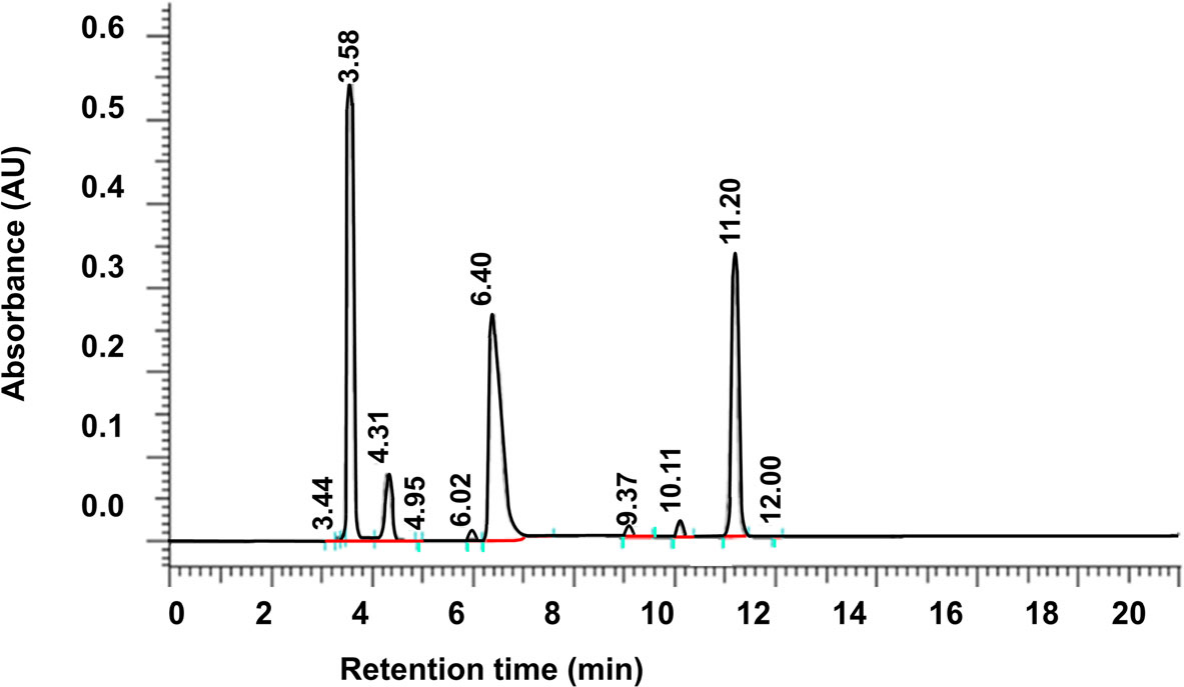
Quantitative determination of vitamins content in TPN admixture (TPN IIB) by HPLC (t_R_ = 3.58 min – ascorbic acid, t_R_ = 4.31 min – thiamine, t_R_ = 6.40 min – pyridoxine)

Quantitative determination of ascorbic acid, thiamine and pyridoxine was performed using HPLC. The initial analytical conditions for the development of the chromatographic method for vitamins were based in methods described in the literature for other purposes [10, 13]. Samples (I-IV) just after preparation (t = 24 h) and after storage for 8 days in a refrigerator with temperature of 4 ± 2 °C (t = 8 days) and next after 24 h of storage in room temperature (21 ± 2 °C) without light protection (t = 8 days +24 h) were analysed. Samples of the admixtures were shaken for 10 min with chloroform in ratio 1:100 and then vortexed for 30 min. Separation of aqueous phase was performed at 40 °C. The isolated aqueous phases were injected onto the chromatographic column in order to determine ascorbic acid, thiamine and pyridoxine concentrations (Fig. 1). The HPLC equipment consisted of a Merck Hitachi L-7100 HPLC pump, a L-7450 photo diode array detector, a L-7200 autosampler, a D-700 interphase module and a column oven. The analytical column was reverse phase C18 LiChrospher 100, 5 μm, 250 × 4 mm. The flow rate was 0.5 ml min − 1 and UV detection was performed at 254 nm for ascorbic acid and at 280 nm for the other vitamins at room temperature. The mobile phase consisted of two solvents: solvent A - 0.05 M NH4H2PO4 (adjusted to pH 3 with orthophosphoric acid), and solvent B – acetonitrile. All solvents were filtered through a 0.45 μm filter and degassed by ultrasonication. The gradient mode was used in the following scheme:

**Table:** 

Time [min]	Solvent A	Solvent B	Flow rate [ml/min]
0 ➔ 3	98%	2%	0.5
3 ➔ 5	95%	5%	0.7
5 ➔ 25	75%	25%	1.0

The method was validated and precision, accuracy, linearity, specificity, detection and quantity limits were established.

### Statistical analysis

All experimental results obtained are presented as mean and standard deviation (SD). Across all time points, the results were evaluated using the non-parametric ANOVA Friedman test. Comparisons between bag type for each composition of TPN admixture (for example IA vs IB) were evaluated using the non-parametric Mann–Whitney U test. Statistica 13 software (StatSoft, Kraków, Poland) was used in all data analysis. Values of *p* < 0.05 were considered statistically significant.

## Results and discussion

The TPN admixtures were prepared following current standards of practice in hospital pharmacy. All admixtures were prepared with trace elements and vitamins. In line with clinical practice, admixtures were stored up to 8 days in refrigerator (without light exposure). All preparations (I-IV) were placed in monolayer EVA bags, with (yellow bags (in built protection), samples B) or without UV protection (transparent bags, samples A). The choice of type bag was dictated by physician’s needs in our collaborating hospital, and represented the real clinical use scenario using commercially available bags.

Our findings provide new and valuable insights into the physicochemical stability of admixtures in monolayer bags which contain mixtures of vitamins and trace elements. In the current literature, the reported studies only focus on light exposure of parenteral nutrition and its chemical stability [12, 17, 18] and there are no reports comparing physical stability of parenteral nutrition in UV protected bags with those without UV protection. It is commonly known that vitamins are sensitive to light exposure, and decomposition starts after 48 h of storage at room temperature and light exposure. Parenteral admixtures are administered to patients only up to 24 h. Therefore, the relevance to clinical practice question posed at the outset of this study was intended to address this pressing question from pharmacists in the hospital Parenteral admixtures are composed of many preparation so each analysis should be carried out for each composition as it is otherwise impossible to determine only four admixtures and infer that those results apply to all admixtures.

The chemical stability of vitamins in TPN admixtures is a very important factor. There may be chemical instability due to interactions between vitamins and microelements mixed in the same bag [13]. Trace elements may promote oxidation of vitamins; for example, copper ions can catalyzed the oxidation reaction of ascorbic acid in the presence of oxygen [11]. This reaction could rapidly occur in monolayer bags which provide less protection from oxidation than multilayered bags. As such, mixing vitamins and trace elements together in one bag seems to be a limitation however it is an approach recommended by parenteral nutrition specialists. On the other hand, lipid emulsions have a role as photoprotector in the TPN admixtures [19], and so the presence of lipid emulsion could compensate in situations where monolayer bags are used.

### Visual inspection

Visual inspection of a completed TPN admixture, which are opaque, is mainly limited by human visual accuracy [10]. Despite its limitations, visual observation is necessary in clinical practice, because this method allows detection of physical instability such creaming or phase separation. Creaming is the initial phase of emulsion breakdown and occurs upon mixing of the lipid emulsion with other components of parenteral nutrition, such as electrolytes or vitamins. The presence of a cream layer normally occurs in parenteral admixtures and is visible at the surface as an opaque white layer separated from the remaining parenteral admixture. Cream layer, since it is reversible, is generally considered safe [20, 21]. Visual inspection of all prepared TPN admixtures did not reveal any changes during storage. For both bag types, very slight creaming, which disappeared after a short period of mixing, was observed in all admixtures after 24 h of storage at room temperature. Creaming occurred in all admixtures, regardless of composition, and so was considered acceptable, in line with clinical practice.

### Microscopic observation

Microscopic observation is a very important method which both facilitates detection of larger oil droplets, ranging between 3 μm and 1 mm and also detection of early flocculation in parenteral admixtures. The disadvantage and limitation of this method is its poor reliability in sizing distributions and calculating a medium droplet size. Consequently, statistical analysis in these sample types is not robust as a rule. Nevertheless, it is not without merit and finds use, as microscopic observation is rapid and easy due to the availability of image analyzing software, and additionally, represents a means of low cost stability assessment. The major technical advantage of this technique is the ability to observe and measure individual particles. However here, combining microscopic observation with other analytical methods was necessary in assessment of the stability of the TPN admixtures, as there were no lipid globules larger than 5 μm.

**Fig. 2:**
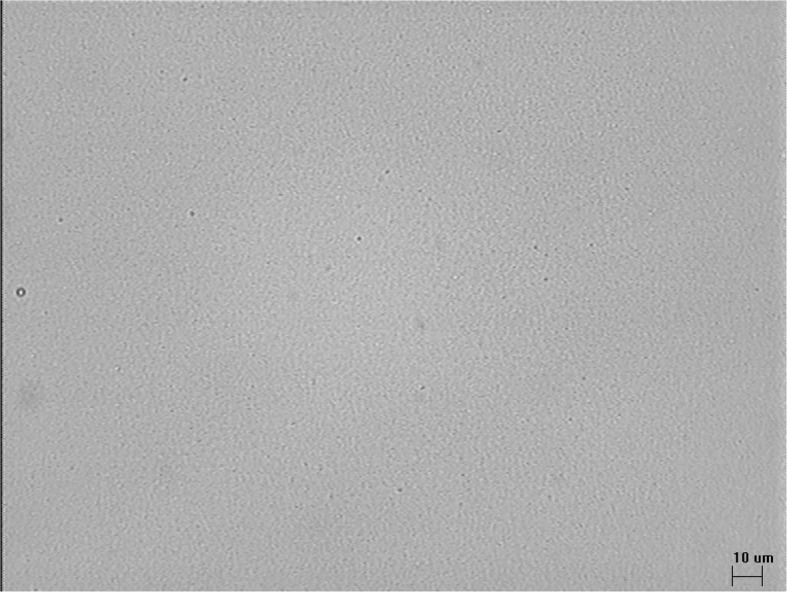
Representative photomicrograph of TPN II after 8 days+24 h of storage

Under microscopic observation, no oil droplets larger than 2 μm were detected in the parenteral admixtures (Fig. 2) suggesting that the parenteral admixtures were stable and could be considered as safe for patients when administered intravenously. Optical microscopy allowed for determination of the higher diameters of lipid globules.

### Determination of lipid droplet size

**Fig. 3:**
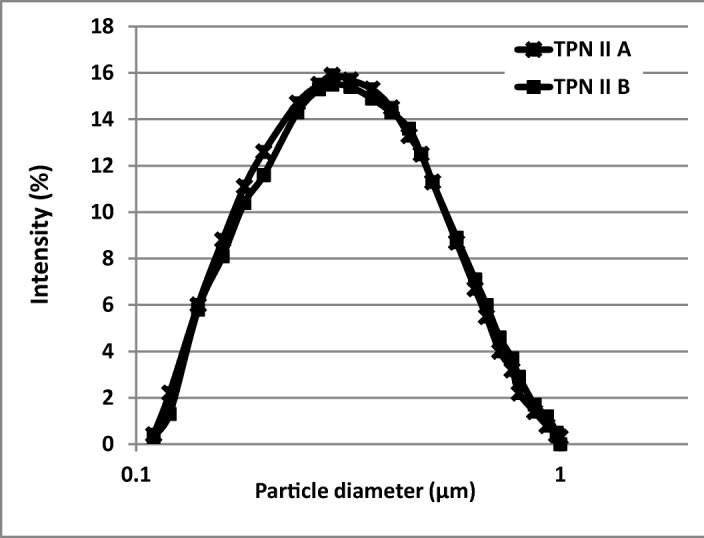
Distribution of lipid droplets of parenteral admixtures taking into account UV protection (LD method)

Table 2 summarizes the median lipid globules (d_0.5_) and d(_0.9_) parameters obtained by LD method, the Z-average obtained by DLS method, and at time points of 0, 24 h, 8 days and 8 days +24 h. Z-Average was seen to be in the range of 230–266 nm, while the polydispersity index (PDI) was 0.114–0.270, which indicates homogeneity of studied admixtures. From baseline (t = 0), no statistically significant changes (*p* < 0.05) in the composition of admixtures were noted in Z-average (± 25 nm) during storage of TPN admixtures, and across both bag types (Fig. 3). The DLS results are in agreement with recommendation of USP 31 <729> which indicates that the mean droplet diameter size obtained by light scattering methods (MDS) must be less than 500 nm for lipid parenteral emulsions.

**Table 2:** Lipid emulsion droplet size (nm **±** SD) within TPN admixtures (LD and DLS method) (*n* = 9; mean ± SD; *p* < 0.05, between 0 and 8 days +24 h and between types of bag)

TPN admixture	d_0.5_ (LD method)				d_0.9_ (LD method)				Z-average (DLS method)			
	t = 0	t = 24 h	t = 8 days	t = 8 days +24 h	t = 0	t = 24 h	t = 8 days	t = 8 days +24 h	t = 0	t = 24 h	t = 8 days	t = 8 days +24 h
IA	330 ± 4.2	320 ± 3.7	320 ± 3.3	330 ± 3.2	570 ± 1.0	560 ± 1.8	570 ± 0.9	570 ± 1.1	250 ± 6.2	245 ± 7.1	252 ± 5.5	252 ± 4.6
IB	310 ± 3.3	320 ± 2.8	330 ± 3.5	320 ± 2.7	560 ± 1.3	560 ± 1.1	560 ± 1.8	560 ± 1.8	240 ± 5.6	249 ± 6.8	245 ± 4.8	244 ± 3.8
IIA	330 ± 3.1	340 ± 3.1	340 ± 5.2	320 ± 2.5	560 ± 1.7	560 ± 2.0	570 ± 1.4	560 ± 1.5	248 ± 4.8	248 ± 5.5	243 ± 4.5	257 ± 5.1^a^
IIB	320 ± 3.2	330 ± 2.9	330 ± 2.6	330 ± 2.0	570 ± 1.6	570 ± 1.2	570 ± 0.8	570 ± 1.3	240 ± 3.7	252 ± 5.9	249 ± 6.1	254 ± 5.5
IIIA	300 ± 4.2	330 ± 3.5	300 ± 1.7	320 ± 3.0	560 ± 2.1	560 ± 1.9	560 ± 1.9	570 ± 2.3	255 ± 3.5	256 ± 4.7	252 ± 4.8	256 ± 4.8
IIIB	310 ± 2.1	310 ± 3.1	300 ± 1.8	300 ± 3.4	570 ± 2.4	560 ± 2.2	570 ± 1.8	560 ± 1.7	252 ± 5.1	254 ± 4.9	253 ± 4.1	258 ± 3.7
IVA	320 ± 2.5	330 ± 4.2	330 ± 2.6	310 ± 2.7	560 ± 1.5	570 ± 1.5	570 ± 0.8	560 ± 1.8	238 ± 5.6	237 ± 3.9	242 ± 3.9	241 ± 4.1
IVB	310 ± 4.0	320 ± 2.2	320 ± 3.1	320 ± 2.2	560 ± 2.5	560 ± 2.0	560 ± 1.6	570 ± 1.9	244 ± 4.8	241 ± 5.1	243 ± 5.0	232 ± 2.9^a^

^a^statistically differences regard t = 0

The median (d_0.5_) of lipid emulsion particles in TPNs was 310–340 nm and 90% of lipid globules (d_0.9_) were under 560–570 nm using LD method (Table 3). No lipid droplets larger than 1 μm were detected in any of the admixtures. No statistically significant changes (*p* < 0.05) were noted in median (d_0.5_) and d_0.9_ parameters during storage of TPN admixtures from t = 0. Moreover, regardless of container type, lipid droplet size did not change during the storage (Fig. 3 2).

**Table 3:** pH and zeta potential values of TPN admixtures (*n* = 9; mean ± SD; *p* < 0.05, between 0 and 8 days+24 h and between types of bag)

TPN admixture	pH				Zeta potential [mV]			
	t = 0	t = 24 h	t = 8 days	t = 8 days+24 h	t = 0	t = 24 h	t = 8 days	t = 8 days+24 h
I A	6.38 ± 0.02	6.36 ± 0.03	6.37 ± 0.02	6.35 ± 0.01	−31 ± 1.7	−29 ± 1.4	−30 ± 1.5	−29 ± 2.0
I B	6.35 ± 0.03	6.33 ± 0.01	6.35 ± 0.03	6.33 ± 0.02	−28 ± 1.5	−27 ± 1.8	−29 ± 1.3	−27 ± 1.7
II A	6.11 ± 0.01	6.09 ± 0.02	6.09 ± 0.04	6.10 ± 0.03	−31 ± 1.3	−30 ± 2.0	−30 ± 1.8	−29 ± 1.4
II B	6.10 ± 0.04	6.08 ± 0.03	6.11 ± 0.01	6.09 ± 0.02	−28 ± 1.9	−31 ± 1.7	−29 ± 1.6	−30 ± 1.1
III A	5.81 ± 0.03	5.79 ± 0.04	5.83 ± 0.02	5.77 ± 0.02	−27 ± 2.1	−26 ± 1.6	−29 ± 1.4	−28 ± 2.0
III B	5.81 ± 0.02	5.80 ± 0.02	5.83 ± 0.03	5.79 ± 0.04	−29 ± 2.0	−30 ± 2.2	−31 ± 1.7	−28 ± 1.5
IV A	5.55 ± 0.04	5.53 ± 0.03	5.56 ± 0.02	5.54 ± 0.02	−27 ± 1.9	−30 ± 1.9^a^	−28 ± 1.3	−28 ± 1.6
IV B	5.58 ± 0.02	5.55 ± 0.02	5.57 ± 0.04	5.56 ± 0.03	−29 ± 1.3	−29 ± 1.8	−31 ± 2.0	−27 ± 1.6

^a^significant differences relative to t = 0

Comparing DLS and LD methods, DLS (Z-average), was approximately 60 nm smaller than the median value recorded using the LD method. It is well known that the more sensitive method which allowed to detect the lipid droplets size below 500 nm is the DLS method, however laser diffraction is valuable as it allows for detection of larger droplets.

### Zeta potential determination

The most important factor in nutrition is that all nutrients be converted into small molecules in the digestive system. A negative zeta potential is required for ingress and egress of nutrients from cells [12]. Lipid emulsions are considered stable if they have negative zeta potential between −20 and − 50 mV [13]. Regardless of the type of the TPN container (bag) and level of UV protection, all of the TPN admixtures studied here were seen to have a zeta potential in range of −26 to −31 mV, thereby indicating their inherent stability. The zeta potential during storage did not undergo statistically significant change (*p* < 0.05) at any storage time point in comparison with time zero and regardless of container type (Table 3).

### Evaluation of pH

The pH values in parenteral admixtures were within a narrow range (5.53–6.38) at time zero [22] and did not experience significant changes (p < 0.05) during storage (Table 3). Admixtures (IV) containing Aminoven Infant 10% as amino acids recorded a slightly lower pH than the other admixtures with other sources of amino acids. It is well known that macronutrients can alter the pH values and even the stability of TPN admixtures [10]. The pH of parenteral admixtures is strongly influenced by the decreasing buffering effect when amino acid concentrations are lower. If the pH of a parenteral admixture is was noted to be lower than 5.0, it could be unstable and unsafe for patient [11, 19]. The pH range for the commercial lipid emulsions used for parenteral admixtures is between 6.0 and 9.0. This pH maintains the negative charge of the oil globules and guarantees the stability of the lipid emulsion [22].

### Quantitative determination of vitamins content

Quantitative determination of ascorbic acid, thiamine and pyridoxine levels was performed using HPLC. The method was validated and the recorded results (Table 4) indicate that this method is specific, linear precise and accurate in the determination of these vitamins in parenteral nutrition admixtures.

**Table 4:** Vitamins Content [% of initial concentration] (HPLC method) (*n* = 9, mean ± SD; *p* < 0.05, between 0 and 8 days+24 h and between types of bag)

TPN admixture	Ascorbic acid			Thiamine			Pyridoxine		
	t = 0	t = 8 days	t = 8 days +24 h	t = 0	t = 8 days	t = 8 days +24 h	t = 0	t = 8 days	t = 8 days +24 h
I A	100	97.81 **±** 0.25	92.31 **±** 0.26^a^	100	98.33 **±** 0.21	93.83 **±** 0.19^a^	100	98.71 **±** 0.22	95.72 **±** 0.23^a^
I B	100	98.54 **±** 0.22	94.11 **±** 0.19^a^	100	99.45 **±** 0.19	95.23 **±** 0.20^a^	100	99.03 **±** 0.24	96.63 **±** 0.25^a^
II A	100	96.88 **±** 0.21	90.12 **±** 0.24^a^	100	99.12 **±** 0.23	94.43 **±** 0.25^a^	100	99.01 **±** 0.18	94.23 **±** 0.21^a^
II B	100	96.96 **±** 0.19	92.66 **±** 0.22^a^	100	99.21 **±** 0.22	96.55 **±** 0.29^a^	100	99.12 **±** 0.22	96.41 **±** 0.19^a^
III A	100	97.01 **±** 0.26	90.05 **±** 0.25^a^	100	98.99 **±** 0.18	93.03 **±** 0.23^a^	100	98.98 **±** 0.27	93.89 **±** 0.29^a^
III B	100	96.96 **±** 0.15	92.55 **±** 0.18^a^	100	99.19 **±** 0.29	95.99 **±** 0.24^a^	100	99.01 **±** 0.24	96.89 **±** 0.23^a^
IV A	100	97.28 **±** 0.22	91.02 **±** 0.21^a^	100	99.22 **±** 0.22	95.89 **±** 0.29^a^	100	99.17 **±** 0.24	95.02 **±** 0.27^a^
IV B	100	97.86 **±** 0.19	93.01 **±** 0.23^a^	100	99.18 **±** 0.25	96.43 **±** 0.27^a^	100	99.66 **±** 0.27	96.94 **±** 0.21^a^

^a^significant differences relative to t = 0

The specificity of the method for assay of the ascorbic acid, thiamine and pyridoxine in the presence of other components of parenteral admixtures was evaluated by the comparison of the chromatograms obtained from a parenteral admixtures containing the standard vitamins in study (C, B2 and B6) with parenteral admixture without the vitamins (placebo). The purity determination of the chromatographic peaks was also used with the software of diode array detector. The specificity of the assay of each vitamins was determined by the comparison between the spectrum of parenteral admixture with the standard of each vitamins, placebo and the parenteral admixture, verifying that the peak observed in the spectrum is attributed to one component alone.

The linearity was evaluated on three different days, against three vitamin standards with five concentration levels, in the ranges of 50–150 mg/ml (vitamin C), 1–5 μg/mL (vitamin B2) and 30–90 μg/mL (vitamin B6). The linearity of the method was determined by linear regression analysis of the values obtained experimentally with the software Excel (Microsoft).

Precision was considered at two levels: repeatability and intermediate precision. It was determined by intra and inter-day assays. Stock solutions of these vitamins were prepared and aliquots were taken to prepare solutions at three levels of concentration: 80%, 100% and 120% of the sample work concentration. For B2 (3, 3.5 and 4.5 μg/mL); B6 (45, 60 and 75 μg/mL) and C (80, 100 and 130 mg). The precision of the method was assessed by the SD and RSD of the values obtained experimentally over three consecutive days. The accuracy of the method was verified by determining the known recovery amount of standard vitamins in the spiked parenteral nutrition placebo. Validation parameters of HPLC method were: precision RDS 1.20, 1.84 and 2.23%, linearity range 1.0–10.0, 80–300, 3.2–16.0, R: 0.9978, 0.9988, 0.9986, LOD (ng/ml) 0.30, 12.0, 1.41, LOQ (ng/ml) 0.99, 39.60, 4.65 for thiamine, ascorbic acid and pyridoxine, respectively. Each sample was analyzed three times.

Data presented in Table 4 show mean and standard deviation (SD) value of the percent content to initial concentration (t = 0) of vitamin C, thiamine and pyridoxine in the parenteral admixtures until 8 days plus 24 h of storage.

No statistically significant variations were observed (*p* < 0.05) in vitamins content through the study comparison with time zero and regardless of container type stored for 8 days at 4 ± 2 °C. Results obtained show statistically insignificant changes in ascorbic acid, thiamine and pyridoxine concentration after 8 days of storage at 4 °C - the observed quantity decrease for those vitamins were below 2% for thiamine and pyridoxine and below 3% for ascorbic acid (Table 4). No differences between samples after preparation (t = 0) and stored for 8 days at 4 ± 2 °C were expected due to the lack of exposure to light. However statistically significant differences (*p* < 0.05) in vitamin content were noticed after 8 days at 4 ± 2 °C plus 24 h at room temperature and light exposure, especially for ascorbic acid (about 9% losses from initial concentration). It is worth noting that these differences were observed for all tested parenteral admixtures despite the UV-protection. Storage of TPN admixtures with vitamin C, thiamine and pyridoxine at room temperature for 24 h resulted in a non-significant (p < 0.05) decrease in vitamins content for each composition of TPN admixtures when UV-protected bags are compared with non-UV-protected bags. Losses were seen to be lower for UV-protected bags but still within the pharmacopoeial range (Table 4). For both bag’s type, the vitamin content remained within the pharmacopoeial range, which means that during storage, the content of the drug (vitamin) was not less than 90% of the declared quantity. Of note, the lack of observed variation between the two types of EVA bags is an interesting and potentially valuable result considering the practical, real use aspects and constraints of TPN administration. Many authors show that concentration of the least stable vitamin, ascorbic acid, in parenteral admixtures decreases significantly after 48 h at room temperature [13, 14] but in practice TPN admixtures are administered to patients up to 24 h after preparation.

No influence of bag type (level of UV-protection) on the physicochemical stability of the investigated TPN admixtures was observed throughout duration of this study. Quantitative analysis of vitamins indicated small differences in their content which depended on storage conditions. The little higher (about 1–2%) decomposition of selected vitamins was observed for non-UV-protected bags, and so confirms that thiamine, pyridoxine and ascorbic acid are sensitive to light. However, despite these losses, levels remained within the pharmacopoeial range. Moreover it should be remembered that the lipid emulsion plays a role of photo-protector in the TPN admixtures, thus its absence facilitates the degradation of photo sensitive vitamins such as B1, B6 and C. Thus, the stability of these vitamins, despite the UV protection, may possibly be increased in formulations containing lipid emulsion.

### Limitations

Our results here shall be valuable in daily, practical and clinical use during which the pharmacist must decide what type of bags are to be used for parenteral nutrition they should use for safe for patients and good price for hospital. UV protected bags are likely to be more expensive with a reviewing of pricing suggesting a difference of approximately 1 Euro per bag, however the final cost would be dependent on supplier and tender conditions.. As such, these results find utility in informing the pharmacist’s choice of parenteral nutrition bag and striking the healthcare economic balance between patient safety with cost.

It is also worth noting that the first analysis, time 0, was undertaken at approximately 12 h after the TPN admixtures were made, due to the time required for sample transportation, under controlled conditions (4 °C), from the collaborating hospital to our department where the analysis took place. Whilst not ideal, this may also reflect normal conditions and time limitations between preparation and administration to the patient. Delaying to start the analysis is a potential limitation, however, according to our previous experience in physical stability of parenteral admixtures [15, 16], it should not have an impact on final results.

The stability of lipid globules is a critical parameter which determines the safety of the therapy and must be verified using analytical methods. Neither European nor Polish Pharmacopeias limit the oil droplet size for parenteral emulsion. However, the United States Pharmacopeia (USP) sets out two limits for globule size distribution; mean droplet size (MDS) of the globules, which should not exceed 500 nm and the percentage of the volume of the large-in-diameter tail of the lipid droplet distribution related to the total lipid volume (PFAT_5_), should not exceed 0.05%. Particle size could be determine by two of pharmacopeia method: Method I (which employs two techniques: 1 and 2) and Method II. Method I (technique 1) is light-scattering techniques (dynamic light scattering, DLS). DLS technique (used in this study) records a Z-average parameter which is the intensity-weighted mean droplet diameter and can be correlated to limits MDS. Method I (technique 2) employs classical light scattering, based on Mie scattering theory (laser diffraction, LD). LD technique (also used in this study) detects oil globule diameters from 1 nm to 5 μm and allows determination of the median of lipid globules (d_0.5_) - the maximum particle diameter below which 50% of sample volume occurs and d_0.9_ parameter - the maximum particle diameter below which 90% of sample volume occurs. Method II is a light obscuration or light extinction – it is a technique called single-particle optical sizing (SPOS) and allowed to determine PFTA_5_ parameter. Method II allows to determine oil droplets above 5 μm. The limitation was that in our laboratory we could not determine PFTA_5_ due to lack of such equipment. However, despite the fact that PFAT_5_, which is mandatory by the US Pharmacopeia, was not used in this study is not a major limitation LD in combination with microscopic observations facilitated the detection of larger globules. Other authors have also suggested this as a potential solution when determination of PFAT_5_ is not possible_._

The limitation of LD and PCS methods is the minimum sample dilution with water before analysis required to obtain proper obscuration. In a situation where lipid globules have agglomerated, sample dilution may disrupt the agglomerates. Hence, it is recommended that microscopic observation, where samples can be assessed without dilution, should always be a part of physical analysis of TPN admixtures [14].

The limitation of zeta potential measurement is choosing the appropriate dilution of samples because zeta potential can vary with the level of dilution. But this method was validated before starting the analysis and we determined that all samples should be diluted 1:100 with water for injection, as in our previous studies [14].

The limitation of quantity analysis of vitamins is fact that studies were carried out after transportation from Cracow to Gdansk (about 24 h after preparation) (t = 0), on the 8th day of storage at 4 ± 2 °C (t = 8 day without light exposure) and again 24 h later (t = 8 day +24 h, under regular light conditions), when TPN admixtures were stored at room temperature. It resulted in that we could not determine the point when decomposition of vitamins occurred. The ideal situation will be that samples are collected and analysis just after preparation, but it was impossible as we described above.

## Conclusion

Our study are valuable in pharmaceutical practice. Parenteral admixtures with vitamins can be safety administered to patient when they are prepared and stored up to 8 days at 4 °C without light exposure plus 24 h at room temperature with light exposure, in monolayer EVA bags without UV-protection. For studied parenteral nutrition compositions, there is no needs to use more expensive EVA bags with UV protection. It is worthy to remember that if we want administer to patient another formula of parenteral admixtures, physicochemical studies should be perform again.
